# Extrinsic functional connectivity of the default mode network in crack-cocaine users

**DOI:** 10.1590/0100-3984.2016.0115

**Published:** 2018

**Authors:** Diego Lima Nava Martins, Talles Destefani de Souza Valiatti, Júlia D'Ávila, Lucas Freire Ferreira, Edson Kruger Batista, Paulo Rodrigo Bazán, Rodrigo Stênio Moll de Souza, Ester Miyuki Nakamura-Palacios

**Affiliations:** 1 MD, Department of Internal Medicine, Health Sciences Center, Graduate Program in Medicine, Federal University of Espírito Santo (UFES), Vitória, ES, Brazil.; 2 Department of Electric Engineering, Technology Center, Brazilian Research Group on Brain and Cognitive Engineering (BRAEN), Federal University of Espírito Santo (UFES), Vitória, ES, Brazil.; 3 MD, MSc, Laboratory of Cognitive Sciences and Neuropsychopharmacology, Graduate Program in Physiological Sciences, Federal University of Espírito Santo (UFES), Vitória, ES, Brazil.; 4 Laboratory for Medical Research 44 (LIM-44-Laboratório de Investigação Médica 44), Department of Radiology, University of São Paulo (USP), São Paulo, SP, Brazil.; 5 MD, MSc, Department of Internal Medicine, Health Sciences Center, Brazilian Research Group on Brain and Cognitive Engineering (BRAEN), Federal University of Espírito Santo (UFES), Vitória, ES, Brazil.; 6 MD, PhD, Graduate Program in Medicine, Laboratory of Cognitive Sciences and Neuropsychopharmacology, Brazilian Research Group on Brain and Cognitive Engineering (BRAEN), Federal University of Espírito Santo (UFES), Vitória, ES, Brazil.

**Keywords:** Substance-related disorders, Magnetic resonance imaging/methods, Brain/physiopathology, Image interpretation, computer-assisted, Functional neuroimaging

## Abstract

**Objective:**

This study aimed to explore the functional connectivity of the default mode network (DMN) in crack-cocaine users, in comparison with that observed in age-matched non-drug-using controls.

**Materials and Methods:**

Inpatient crack-cocaine users who had been abstinent for at least four weeks and age-matched non-drug-using controls underwent resting state functional magnetic resonance imaging. Images were acquired while the subjects rested with their eyes closed. After data preprocessing, DMNs were defined by spatial independent component analysis and seed-based correlation analysis, by chosen regions of interest centered in the ventral anterior cingulate cortex and in the posterior cingulate cortex.

**Results:**

The functional connectivity of the DMN determined by independent component analysis did not differ between the crack-cocaine users and the controls. However, the seed-based correlation analysis seeking a single metric of functional connectivity between specific brain regions showed that the negative connectivity between the ventral anterior cingulate cortex and the left superior parietal lobule was significantly greater in the crack-cocaine users than in the controls.

**Conclusion:**

The results suggest that selective extrinsic network connectivity of the DMN related to motor and executive function is impaired during crack-cocaine addiction.

## INTRODUCTION

Crack-cocaine has emerged as a substance that readily supplies the active principle (cocaine) through the inhalation of smoke from burning rocks of the substance, typically by means of a pipe^(^^1^^)^. How it affects the nervous system remains unknown. However, certain neural circuits are supposedly involved in the establishment and maintenance of drug addiction^(^^2,3^^)^. According to Volkow et al.^(^^4^^)^, there are four interrelated circuits that are involved in or affected by drug addiction: the reward circuit-involving several nuclei in the basal ganglia, notably the nucleus accumbens of the ventral striatum, and relaying information to the ventral pallidum; the motivation/drive circuit-involving the orbitofrontal and subcallosal cortices, dorsal striatum, and motor cortex; the learning/memory circuit-involving the amygdala and the hippocampus; and the control circuit-which controls cognitive flexibility and planning, involving the dorsolateral prefrontal cortex, anterior cingulate cortex, and inferior frontal cortex. Many of these brain structures are associated with the default mode network (DMN)^(^^5-7^^)^, one of the brain networks seen in resting state functional magnetic resonance imaging (rs-fMRI) studies^(^^8^^)^. The DMN is composed of brain regions that are typically deactivated during task demands but exhibit synchronous low frequency oscillation in the resting state^(^^6,7,9^^)^. It is believed that the DMN is related to attention and self-monitoring^(^^10^^)^, or, more precisely, the general monitoring of sensory information associated with the posterior cingulate and adjacent precuneus, as well as the evaluation of the salience of this information together with the medial and orbitofrontal cortices^(^^9^^)^. This provides a new perspective on brain function by addressing the importance of ongoing or intrinsic activity^(^^7^^)^.

Previous rs-fMRI studies have identified changes in functional connectivity in individuals who abuse or are addicted to drugs such as cocaine, heroin, morphine, nicotine, alcohol, and caffeine. However, to our knowledge, there have been no such studies involving individuals addicted to crack-cocaine. We hypothesized that the DMN would be affected in crack-cocaine users. Therefore, this study aimed to explore the functional connectivity of the DMN by using independent component analysis (ICA) and seed-based correlation analysis of rs-fMRI data collected from crack-cocaine users and age-matched non-drug-using controls.

## MATERIALS AND METHODS

For this study, we interviewed 38 individuals with crack-cocaine addiction, as defined in the Diagnostic and Statistical Manual of Mental Disorders, fourth edition (DSM-IV), recruited from among those under treatment at a mental health clinic for drug dependence treatment in the state of Espírito Santo, Brazil. Of those 38 individuals, 10 met the inclusion criteria. At the mental health clinic, the subjects were evaluated in a highly restricted and controlled environment. Urine samples were collected for drug testing, and all of the subjects tested negative. We acquired rs-fMRI scans in a 1.5 T scanner at the Cassiano Antônio de Moraes University Hospital, operated by the Federal University of Espírito Santo. Because of technical problems during the acquisition of the images, data for two of the subjects were excluded from the analysis. Therefore, data for eight crack-cocaine users were included in all analyses.

As a control group, healthy, non-drug-using, aged-matched male subjects were recruited from among the employees of the University Hospital and of the private clinic, as well as from among students at the Federal University of Espírito Santo. The rs-fMRI scans of the control group subjects were acquired in the same scanner as were those of the study group.

The inclusion criteria for the crack-cocaine user group were as follows: being male; being over 18 years of age; meeting the criteria for a clinical diagnosis of crack-cocaine dependence, as defined in the symptom checklist for mental disorders of the International Classification of Diseases, 10th revision, and in the DSM-IV; being in stable clinical condition (not requiring hospitalization); being able to read, write, and speak Portuguese; and presenting with no signs or symptoms of severe withdrawal at baseline.

Subjects who were intoxicated with or in withdrawal from a substance other than crack-cocaine were excluded, as were those who had been diagnosed with a psychiatric or physical disorder, including substance abuse disorder and addiction to any substance other than crack-cocaine, nicotine, or caffeine. We also excluded subjects who had been diagnosed with epilepsy or convulsive disorder; those who had experienced delirium tremens during abstinence from crack-cocaine use; those with a history of drug hypersensitivity or adverse reactions to diazepam, other benzodiazepines, or haloperidol; those presenting with any contraindication for MRI, such as electronic implants, metal implants, and claustrophobia, as well as permanent makeup or tattoo received within the last three months; and those with vascular, traumatic, inflammatory, or neoplastic processes detectable by MRI. It should be stated that medications were given to the crack-cocaine users to relieve the symptoms of anxiety and depression that can arise during abstinence.

The study was approved by the Institutional Review Board of the Federal University of Espírito Santo (CAAE Protocol Nos. 19403713.6.0000.5060 and 13528213.2.0000.5060). The study procedures were in strict adherence to the Declaration of Helsinki and in accordance with the ethical standards established by the Committee on Human Experimentation of the Federal University of Espírito Santo, where the study was conducted. All participating subjects gave written informed consent.

We assessed ten inpatients diagnosed with crack-cocaine dependence and eight non-drug-using controls. To do so, we used a brief structured interview designed to obtain sociodemographic data and information regarding the characteristics of drug use.

### MRI

The rs-fMRI scans were obtained while subjects rested quietly, with their eyes closed but awake. The images were acquired in 1.5 T MRI scanners (Achieva; Philips Medical Systems, Best, the Netherlands), with a specific 8-channel sensitivity encoding head coil (SENSE; Philips Medical Systems), at the radiology center of the Cassiano Antônio de Moraes University Hospital.

Functional images were acquired using a gradient echo echo-planar imaging sequence-repetition time/echo time (TR/TE): 3000/50 ms; flip angle = 90º; 35 axial slices; matrix: 64 × 64; field of view = 230 mm; and voxel size: 3.59 × 3.59 × 4.0 mm-with an echo planar imaging factor of 39 and an acquisition time of 10 min 9 s per subject. For registration purposes, a T1-weighted anatomical image was also acquired: TR/TE: 8.8/4.05 ms; flip angle = 80º; matrix: 240 × 240; voxel size: 1 × 1 × 1 mm; 180 axial slices; field of view = 240 mm; and acquisition time = 1 min 28 s.

**Figure 1:**
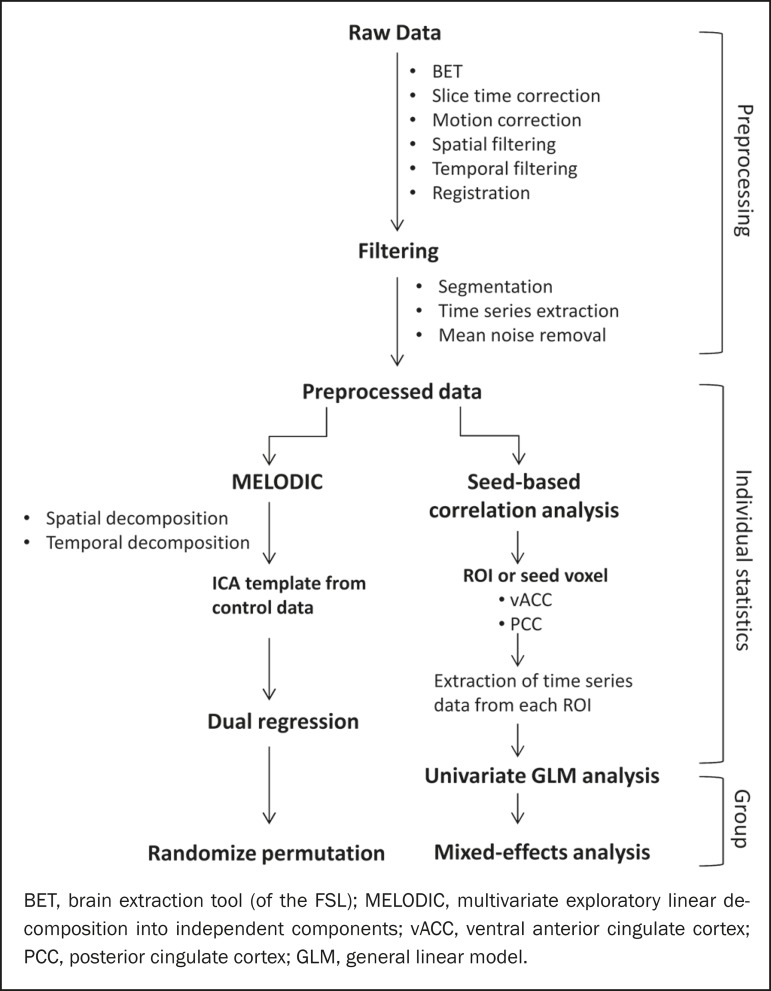
Flow chart of the data analysis process.

Functional images were pre- and post-processed with the Functional Magnetic Resonance Imaging of the Brain software library (FSL, version 5.0.8; FMRIB Analysis Group, Oxford University, UK). In brief, traditional pre-processing steps were performed: head motion, by realigning each volume to the middle volume; slice-timing correction; non-brain material extraction; spatial smoothing, using a 6 mm (full width at half maximum) kernel; high-pass temporal filtering (0.01 Hz); and normalization to the Montreal Neurological Institute standard-space template (2-mm resolution). In addition, to reduce physiological noise, tissues were segmented by using the structural T1-weighted images from each subject. The average white matter and cerebrospinal fluid signals were filtered using linear regression, the residuals of that analysis being used in the subsequent steps (Figure 1).

#### ICA and dual regression

Images were spatially and temporally decomposed in a four-dimensional (time × voxels) data matrix into a set of time courses and associated spatial maps with the multivariate exploratory linear decomposition into independent components interface of the FSL (Figure 1). This instrument was used as a primary approach to objectively identify resting state networks, especially the DMN for this study purpose^(^^11,12^^)^. We performed two separate group ICA runs concatenating the time series of the individuals in each group.

For dual regression analysis (Figure 1), the set of spatial maps from the control group ICA was used as the template, as described by Rytty et al.^(^^13^^)^, to generate subject-specific versions of the spatial maps, and associated time series^(^^11-14^^)^ were used in order to test for voxel-wise group differences. We adopted a nonparametric test, using the "randomize" permutation-testing tool of the FSL and calculating the maximum of 5,000 permutations, with a threshold of *p* = 0.05 corrected for multiple comparisons throughout the brain.

Seed-based correlation analysis

The seed-based correlation analysis employed the same input data employed in the ICA^(^^14,15^^)^. The difference was that the seed-based correlation analysis was based on regions of interest (ROIs), as shown in Figure 1. The chosen ROIs and their Talairach coordinates were based on those that Greicius et al.^(^^5^^)^ defined as the main regions constituting the DMN. Thus, two ROIs (each 8 mm in diameter) were chosen, one centered in the posterior cingulate cortex (Talairach coordinates: x = 2, y = −51, z = 27) and the other centered in the ventral anterior cingulate cortex (Talairach coordinates: x = 2, y = 38, z = −2). For each participant, the signal extracted from those seed regions were input into two separate whole-brain analyses (one for each seed), allowing the positive and negative correlations to be evaluated. Correlation scores were converted to *z*-scores using Fisher's *z*-transformation. We performed group-level analyses using the mixed-effects model implemented in the fMRI Expert Analysis Tool of the FSL, adopting a voxel threshold of *z* > 2.3 and correcting for multiple comparisons at the cluster level using Gaussian random field theory with a cluster significance threshold of *p* = 0.05.

## RESULTS

Baseline sociodemographic characteristics and patterns of drug use are presented in Table 1. All of the crack-cocaine users were young (mean age, 29.1 ± 10.6 years), were male, typically had a low level of education (fewer than three years of schooling), were mostly unemployed, and were single. In addition, most of them were cigarette smokers. With the exceptions of age and marital status, the sociodemographic characteristics (including level of education, employment status, and tobacco use) differed between the crack-cocaine user group and the control group. Such differences were expected considering the impoverishment due to crack-cocaine addiction. On average, the subjects in the crack-cocaine user group had started using crack-cocaine at 22.6 ± 8.9 years of age, consumed 14.8 ± 16.2 rocks per day, and had been abstinent for at least four weeks prior to the beginning of the experimental protocol (Table 1). All of the urine samples collected during the study period tested negative.

**Table 1:** Sociodemographic characteristics and patterns of crack-cocaine use in a sample of male drug users, abstinent for at least four weeks, who underwent rs-fMRI in a 1.5 T scanner (n = 8), in comparison with age-matched non-drug-using male control subjects who also underwent rs-fMRI (n = 8).

Characteristic	Non-drug-using controls	Crack-cocaine users	Statistic	P
Age, in years, mean (SD)	31.4 (7.0)	29.1 (10.6)	t(14) = 0.50	0.62
Years of schooling, mean (SD)	12.7 (3.3)	1.9 (0.8)	t(13) = 9.14	< 0.0001
Employment situation, n (%)				
Formal job	6 (75%)	0 (0%)	χ^2^ = 14	0.016
Informal job	1 (12.5%)	0 (0%)		
Unemployed	0 (0%)	4 (50%)		
Self-employed	0 (0%)	2 (25%)		
On disability	0 (0%)	1 (12.5%)		
Not reported	1 (12.5%)	1 (12.5%)		
Marital status, n (%)				
Single	5 (62.5%)	6 (75%)	χ^2^ = 2.09	0.35
Married	3 (37.5%)	1 (12.5%)		
Divorced	0 (0%)	1 (12.5%)		
Tobacco use, n (%)				
Yes	0 (0%)	5 (62.5%)	Fisher's	0.013
No	8 (100%)	3 (37.5%)		
Crack-cocaine use				
Age, in years, at onset, mean (SD)		22.6 (8.9)		
Amount consumed (rocks/day), mean (SD)		14.8 (16.2)		

### rs-fMRI

#### ICA

The ICA applied to the rs-fMRI data satisfactorily determined the component representing the DMN individually for subjects in the control and crack-cocaine user groups, accurately providing the mean for each group. Neither the dual regression analyses considering the control group as the template nor the randomized permutation analyses identified any differences between the two groups.

#### Seed-based correlation analysis

**Figure 2:**
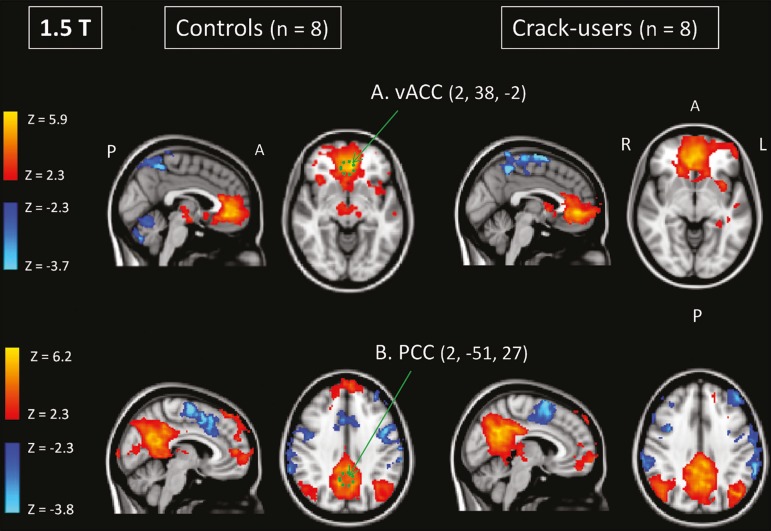
Functional connectivity determined by seed-based correlation analysis centering on ROIs in an 8-mm diameter circle (depicted in green). In **A** the ventral anterior cingulate cortex (vACC: x = 2, y = 38, z = −2) and in **B** the posterior cingulate cortex (PCC: x = 2, y = −51, z = 27), considering the coordinates used by Greicius et al.(5), on rs-fMRI scans of crack-cocaine users and age-matched non-drugusing controls, obtained in 1.5 T scanners. (Red/yellow = positive correlation; blue/light blue = negative correlation).

**Figure 3:**
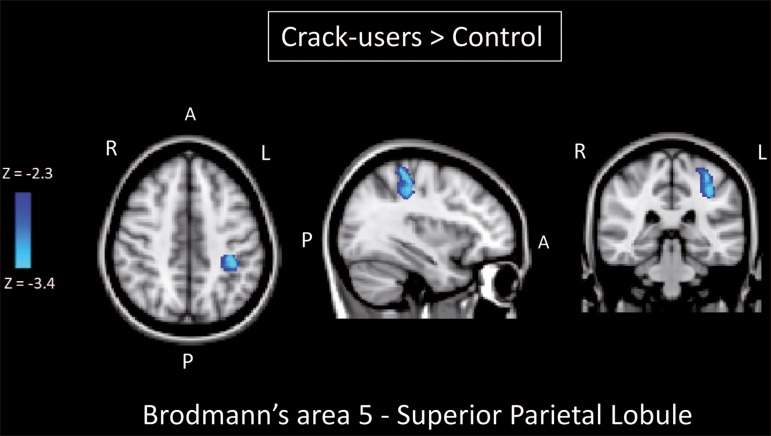
Group-level comparison of negative correlations between crackcocaine users and age-matched nondrug- using controls determined by seed-based correlation analysis of the ROI centered in the ventral anterior cingulate cortex (x = 2, y = 38, z = −2) and the region (x = −32, y = −38, z = 60) corresponding to the left superior parietal lobule (Brodmann’s area 5). The negativity was greater (p < 0.0322) in the crack-cocaine users when compared to the non-drug-using control subjects. From left to right: axial, sagittal and coronal planes. (Blue/light blue = difference of negative correlation).

Activities in the ventral anterior cingulate cortex (ROI centered at x = 2, y = 38, z = −2) and in the posterior cingulate cortex (ROI centered at x = 2, y = −51, z = 27), as depicted in Figure 2, were found to be positively and negatively related to the structures listed in Tables 2 and 3, respectively. No differences were found between the control and crack-cocaine user groups regarding the positive correlations. However, the Figure 3 shows the negative correlation between the ventral anterior cingulate cortex and the region corresponding to the left superior parietal lobule (Brodmann's area 5). As can be seen in Table 3, that correlation was greater in the crack-cocaine user group than in the control group (*p* < 0.0322).

**Table 2:** Regions positively related to ROIs (8 mm in diameter) centered in the ventral anterior cingulate cortex (Talairach coordinates: x = 2, y = 38, z = -2) and posterior cingulate cortex (Talairach coordinates: x = 2, y = -51, z = 27) in crack-cocaine users (n = 8) and age-matched non-drug-using controls (n = 8).

ROI	Group	Cluster	Voxels	P	Max. z	Talairach coordinates			Brodmann's area
						x	y	z	
Ventral anterior cingulate cortex	Control	1	966	0.00152	3.89	-56	-12	-16	21 (left middle temporal gyrus)
		2	7,058	1.64 × 10^-16^	5.61	-6	40	-8	10 (left medial frontal gyrus)
	Crack-cocaine	1	998	0.0012	3.87	-48	-2	-26	20 (left inferior temporal gyrus)
		2	8,499	7.68 × 10^-19^	5.97	2	44	-4	32 (right dorsal anterior cingulate)
Posterior cingulate cortex	Control	1	675	0.0268	4.39	56	0	-22	21 (right middle temporal gyrus)
		2	1,166	0.000885	4.27	-8	66	6	10 (left superior frontal gyrus)
		3	2,781	1.19 × 10^-7^	4.39	30	28	58	6 (right middle frontal gyrus)
		4	13,250	1.63 × 10^-23^	5.95	-4	-52	28	31 (left dorsal posterior cingulate)
	Crack-cocaine	1	1,043	0.00197	4.26	68	-2	-10	21 (right middle temporal gyrus)
		2	1,532	9.45 × 10^-5^	4.77	0	64	4	10 (left medial frontal gyrus)
		3	1,645	4.92 × 10^-5^	5.61	-46	-70	34	39 (left angular gyrus)
		4	1,824	1.8 × 10^-5^	4.11	-36	22	52	8 (left superior frontal gyrus)
		5	15,337	4.92 × 10^-26^	6.26	-2	-52	24	31 (left dorsal posterior cingulate)

**Table 3:** Regions negatively related to ROIs (8 mm in diameter) centered in the ventral anterior cingulate cortex (Talairach coordinates: x = 2, y = 38, z = -2) and posterior cingulate cortex (Talairach coordinates: x = 2, y = -51, z = 27) in crack-cocaine users (n = 8) and age-matched non-drug-using controls (n = 8).

ROI	Group or comparison	Cluster	Voxels	P	Max z	Talairach coordinates			Brodmann's area or region
						x	y	z	
Ventral anterior cingulate cortex	Control	1	581	0.0334	3.46	-22	2	70	6 (left superior frontal gyrus)
		2	1,643	1.55 × 10^-5^	3.79	22	2	62	6 (right superior frontal gyrus)
		3	4,675	2.85 × 10^-12^	3.52	2	-74	-38	Right inferior semilunar lobule
		4	4,756	1.99 × 10^-12^	3.64	-24	-64	46	7 (left superior parietal lobule)
	Crack-cocaine	1	12,432	1.31 × 10^-24^	4.42	-2	-10	72	6 (left medial frontal gyrus)
	Group-level analysis								
	Crack-cocaine > control	1	585	0.0322	3.66	-32	-38	60	5 (left superior parietal lobule)
Posterior cingulate cortex	Control	1	655	0.0312	3.94	-34	44	34	9 (left middle frontal gyrus)
		2	9,727	6.21 × 10^-19^	4.52	2	14	50	6 (right superior frontal gyrus)
	Crack-cocaine	1	2,049	5.3 × 10^-6^	4.53	58	-28	30	40 (right inferior parietal lobule)
		2	2,381	9.54 × 10^-7^	4.54	-42	56	22	10 (left middle frontal gyrus)
		3	2,719	1.79 × 10^-7^	4.49	30	48	26	10 (right superior frontal gyrus)
		4	6,607	2.21 × 10^-14^	4.46	-64	-36	30	40 (left inferior parietal lobe)

## DISCUSSION

In this study, we used ICA and seed-based correlation analysis to evaluate the functional connectivity of the DMN in crack-cocaine addicts. The seed-based correlation analysis showed that the negative or antiphasic relationship between the medial frontal site of the DMN and the superior parietal lobule was stronger in crack-cocaine addicts who had abstinent for at least four weeks than it was in age-matched non-drug-using control subjects.

Exploring the DMN and its connectivity in drug addiction has been of great interest to help improve the understanding of this complex disease^(^^16^^)^. Studies employing rs-fMRI to investigate the effects or consequences of cocaine dependence have produced mixed results, from hyperconnectivity of the anterior cingulate cortex^(^^17^^)^ to decreases in connectivity between the DMN and other networks^(^^18-21^^)^.

The precise brain function supported by the DMN remains unknown. According to Raichle^(^^6^^)^, the DMN supports processes related to emotional processing (involving the ventral medial prefrontal cortex), self-referential mental activity (involving the dorsal medial prefrontal cortex), and the recollection of prior experiences (involving posterior elements of the DMN). The DMN is a functioning network that is never inactive; its level of activity seems to vary only according to the level of consciousness^(^^22,23^^)^. It remains active during mild sedation^(^^24^^)^, anesthesia^(^^25^^)^, and even during a vegetative state^(^^26^^)^, although with some variations of its connectivity^(^^23^^)^, and is completely silent only in brain death^(^^26^^)^. Therefore, it seems reasonable to consider that the DMN would be affected only under extremely life-threatening conditions, which fortunately might not be the case for crack users. In that sense, it is heartening to have found that the overall DMN connectivity in crack users is not yet affected in comparison with that observed in age-matched non-drug-using controls. Consequently, although the use of crack is highly detrimental to our young patients in many ways, there is hope that there will be a good recovery, given that intrinsic brain functioning is preserved or re-established after abstinence.

Compulsive cocaine use has been associated with a balance between increased striatal-anterior prefrontal/orbitofrontal connectivity and decreased striatal-dorsal anterior cingulate connectivity^(^^27^^)^. In the present study, the seed-based correlation analysis showed that negative connectivity between the ventral anterior cingulate cortex and the left superior parietal lobule was greater in crack-cocaine users. This brain area, corresponding to Brodmann's area 5, is situated immediately posterior to the primary somatosensory areas (postcentral gyrus), anterior and to the right of Brodmann's area 7. The superior parietal lobule has been proven to be necessary for the executive rearrangement of information in working memory^(^^28^^)^, impairment of the connectivity between the medial frontal region and the superior parietal lobule observed here in crack-cocaine users, thus potentially being strictly related to the severe executive dysfunction typically seen in crack-cocaine dependent subjects^(^^29^^)^, a condition that aggravates and maintains the drug addiction^(^^2,3,30^^)^. Albeit intriguing, this result needs to be interpreted with great caution and confirmed in a larger sample, especially considering that the veracity of the "negativity" between network relationships is highly uncertain and is still a matter of debate^(^^31^^)^.

This study has certain limitations. We included data from a small number of subjects. Although data from a larger sample were collected, many of the subjects had to be excluded because of technical issues such as artifacts and large head movements. This study was focused only on the analysis of the DMN as the principal resting state network of interest. Therefore, in this exploratory study, the total DMN functional connectivity determined by ICA was found to be preserved in crack-cocaine dependent subjects who had been abstinent for at least four weeks in comparison with that observed for age-matched non-drug-using controls. However, the negative connectivity between the superior parietal lobule and the ventral anterior cingulate cortex in crack-cocaine users was greatest when we performed seed-based correlation analysis with a single metric of functional connectivity. Our results suggest that, in crack-cocaine addiction, the DMN is intrinsically unaffected, although there might be restricted functional connectivity with a region extrinsically related to the DMN.
